# Medical Cannabis Is Not Associated with a Decrease in Activities of Daily Living in Older Adults

**DOI:** 10.3390/biomedicines11102697

**Published:** 2023-10-03

**Authors:** Ran Abuhasira, Lihi Schwartz, Victor Novack

**Affiliations:** 1 Clinical Research Center, Soroka University Medical Center, Be’er-Sheva 8410501, Israel; ranabu@post.bgu.ac.il; 2 Faculty of Health Sciences, Ben-Gurion University of the Negev, Be’er-Sheva 8410501, Israel; 3 Clalit Health Services, Department of Family Medicine, Dan-Petah Tikva District, Petah Tikva 5239530, Israel; lihi.shwartzh@gmail.com; 4 Sackler Faculty of Medicine, Tel Aviv University, Tel-Aviv 6139001, Israel

**Keywords:** older adults, medical cannabis, marijuana, functional status, opioids

## Abstract

The proportion of older adults using medical cannabis is rising. Therefore, we aimed to assess the effects of herbal medical cannabis on the functional status of older adults. We conducted a prospective observational study of patients aged 65 years or older that initiated cannabis treatment for different indications, mostly chronic non-cancer pain, during 2018–2020 in a specialized geriatric clinic. The outcomes assessed were activities of daily living (ADL), instrumental activities of daily living (IADL), pain intensity, geriatric depression scale, chronic medication use, and adverse events at six months. A cohort of 119 patients began cannabis treatment: the mean age was 79.3 ± 8.5 and 74 (62.2%) were female. Of the cohort, 43 (36.1%) experienced adverse effects due to cannabis use and 2 (1.7%) required medical attention. The mean ADL scores before and after treatment were 4.4 ± 1.8 and 4.5 ± 1.8, respectively (*p* = 0.27), and the mean IADL scores before and after treatment were 4.1 ± 2.6 and 4.7 ± 3, respectively (*p* = 0.02). We concluded that medical cannabis in older adults has a number of serious adverse events, but was not associated with a decrease in functional status, as illustrated by ADL and IADL scores after six months of continuous treatment.

## 1. Introduction

While cannabis has historically been predominantly associated with recreational use among younger demographics, recent shifts in societal attitudes and evolving legalization efforts have sparked a surge of interest in exploring its potential effects on older populations [1,2]. Medical cannabis, often without a comprehensive understanding of its safety and effectiveness, is now being used for various conditions, including but not limited to chronic pain, multiple sclerosis, chemotherapy-induced nausea and vomiting, Parkinson’s disease, epilepsy, and others [3,4,5,6]. The protocols for cannabis treatment in older adults still differ between countries and between practitioners due to the differences in the composition of cannabis medicines and the different regulations [7,8].

Among older adults, cannabis exhibits its effectiveness across multiple dimensions, notably in mitigating pain, enhancing mood, refining sleep quality, and elevating overall quality of life. Furthermore, the therapeutic introduction of medical cannabis yields the advantageous outcome of facilitating decreases in opioid dosages or utilization, which is especially pertinent within the context of chronic pain management [9,10,11]. It is crucial to acknowledge that safety constitutes a paramount consideration within this demographic. The nuanced intricacies encompassing older adults bring to the forefront heightened apprehensions, encompassing augmented susceptibilities to falls, visual attention, and plausible cardiovascular ramifications [12,13,14].

Functional status, which is a crucial aspect of geriatric care, plays a significant role in assessing overall well-being. As the global population continues to age, the focus on improving the quality of life of older adults becomes increasingly pertinent. Activities of Daily Living (ADLs), encompassing essential self-care tasks such as bathing, dressing, eating, and mobility, serve as critical indicators of an individual’s functional independence and overall well-being. With the rise in chronic conditions and age-related ailments, finding innovative therapeutic approaches that enhance older adults’ ability to perform ADLs is of paramount importance. Moreover, a decline in function can lead to heightened healthcare utilization, diminished quality of life, compromised independence, and an elevated risk of mortality. The Katz ADL scale and the Lawton instrumental activities of daily living (IADL) scale are well-known scales that assess functional status in older adults [15,16].

The impact of medications on IADL and ADL scores is a critical consideration in geriatric healthcare. Polypharmacy, often necessary to manage the multiple chronic conditions prevalent in older adults, has been associated with functional impairment [17]. Some medications, particularly those with sedative or psychotropic properties such as benzodiazepines and anticholinergics, can potentially compromise cognitive and physical functioning, leading to difficulties in performing IADL and ADL tasks [17]. Conversely, appropriate medication management can significantly contribute to maintaining or enhancing functional independence. Certain medications, such as those targeting pain, inflammation, or cardiovascular health, can alleviate symptoms that might otherwise impede individuals’ ability to engage in routine tasks, as was shown for angiotensin-converting enzyme inhibitors, for example [18]. Moreover, medication-related adverse effects can exacerbate functional decline, as adverse events can lead to hospitalizations or further limitations in daily activities. The effects of medical cannabis on functional status in older adults are under researched.

With advancing age, alterations in appetite and nutritional status pose significant challenges for older adults [18]. The interaction between cannabis and appetite is mediated primarily by tetrahydrocannabinol (THC) through its effect on the cannabinoid receptor type 1 (CB1). This receptor activation, alongside hormonal shifts and altered sensory perception, collectively shapes cannabis-induced changes in appetite [19]. The modulation of appetite by cannabis has practical significance, notably in individuals undergoing chemotherapy or confronting conditions characterized by appetite loss, such as HIV/AIDS [20,21]. By enhancing appetite, cannabis can potentially counteract weight loss and malnutrition in medically compromised individuals.

We previously published the treatment protocol and initial data about the cohort of older adults treated with cannabis in a specific clinic [8]. The aim of this prospective study was to assess the effects of herbal medical cannabis on the functional status of older adults following the published protocol of treatment. Our hypothesis was that cannabis use will not decrease functional status after six months of continuous use. Our hypothesis was based on previous studies showing relatively high satisfaction rates from cannabis use in older adults [10], for which we assumed that functional status could be one of the contributing components.

## 2. Materials and Methods

### 2.1. Study Design and Population

The study was conducted in a specialized geriatric clinic aimed to provide medical cannabis therapy within a comprehensive geriatric platform. This was a prospective observational study that included all patients above the age of 65 years who began their cannabis treatment in the clinic during the years 2018–2020. There were no exclusion criteria. The recruitment for the study was stopped due to the emergence of the coronavirus disease-19 (COVID-19) pandemic. The patients were followed for six months from cannabis treatment initiation. The cannabis for patients was supplied by pharmacies in Israel according to the doctor’s prescription and not by the study personnel. The constituents of the cannabis products are reported to the Ministry of Health and include the content of THC and CBD in each product.

### 2.2. Data Collection and Outcomes

Patients were invited to take part in the study and were requested to complete the study questionnaire during their initial appointment, prior to commencing cannabis treatment. Subsequently, they were asked to respond to the same set of study questions after undergoing six months of continuous cannabis treatment. The data collected before treatment included demographics, chronic medications, indication for cannabis use, type of cannabis selected, dosing, and route of administration. In addition, the patients responded to the following questionnaires before and after treatment: Geriatric Depression Scale (GDS) [22], Katz Activities of Daily Living (ADL) [15], Lawton Instrumental Activities of Daily Living (IADL) [16], and Visual Analogue Scale (VAS) for Pain [23]. After treatment patients were also asked to report the following parameters:

- Perception of the general effect of cannabis—global assessment using the Likert scale with seven options: significant improvement, moderate improvement, slight improvement, no change, slight deterioration, moderate deterioration, or significant deterioration.
- Changes in appetite—global assessment using the Likert scale with six options: significant increase, slight increase, no change, slight decrease, significant decrease, or change only immediately after cannabis consumption.
- Adverse effects—incidence, duration, severity, and the need to seek medical care for the reported adverse event.
- Changes in chronic drug regimens—drugs that were added, stopped, and changed in dosing.
- Stopping use of medical cannabis—patients that stopped using cannabis after six months were asked about the reasons for stopping treatment.

### 2.3. Statistical Analysis

Continuous variables with normal distribution were presented as means with standard deviation. Ordinary variables or continuous variables with non-normal distribution were presented as medians with an interquartile range (IQR). Categorical variables were presented as counts and percent of the total. Univariable comparisons were made using paired Student’s *t*-test or paired Wilcoxon test to compare quantitative and ordinal variables, respectively. We used multivariable logistic regression to assess the factors associated with improvements in the different scores assessed in the study. The variables inserted in the model were those significant in univariable comparisons, the basic demographic characteristics, and the data about cannabis use. THC-rich strains were defined as strains with 10% THC content or higher. The definition of non-THC-rich strains included cannabidiol (CBD)-rich strains and patients treated with equal amounts of THC and CBD.

A *p*-value of 0.05 or less (two-sided) was considered statistically significant. SPSS software (IBM, Armonk, NY, USA), version 25.0, was used for statistical analysis.

## 3. Results

We prospectively followed 119 patients between 2018–2020; the patients’ mean age was 79.3 ± 8.5, and 74 (62.2%) were female. Table 1 shows that the most common indication for cannabis treatment was non-specific chronic pain (47.9%) and the main route of administration was tincture (60.5%). The composition of cannabis strains that were used by the patients was varied, containing different proportions of THC and CBD. Of the cohort, 43 (36.1%) experienced adverse effects due to cannabis use; 9 (7.6%) self-defined them as severe and 2 (1.7%) needed medical care. Two patients (1.7%) had died by the end of the follow-up period.

**Figure 1:**
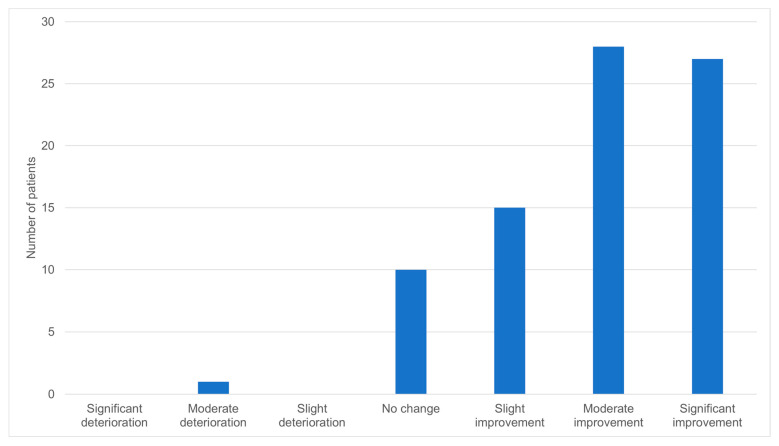
Perception about the general effect of cannabis on the patient’s condition (N = 81).

**Figure 2:**
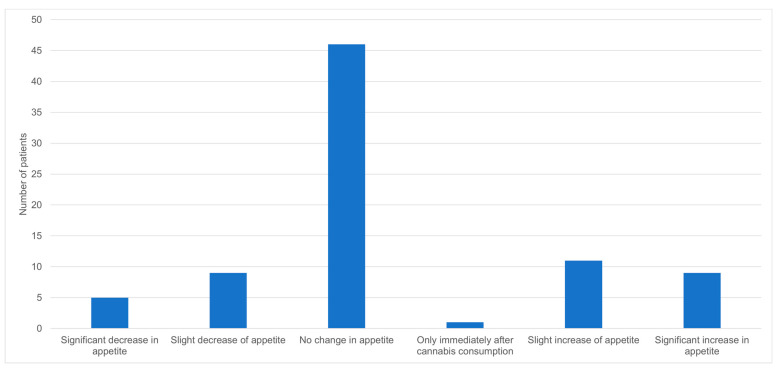
Effect of cannabis on appetite (N = 81).

The effect of cannabis on the general condition of the patients was favorable for most of them, with 70 (86.4%) of the respondents reporting some degree of improvement (Figure 1). Similarly, the effect of cannabis on pain reduction was significant, with a mean reduction of 3.3 on a visual analog scale of 0–10 (Table 2, *p* < 0.001). In contrast, 46 (56.8%) of the respondents did not have any change in their appetite, 14 (17.3%) had a decrease in their appetite, and 20 (24.7%) had an increase in their appetite (Figure 2). After using cannabis for six months, 30 patients (25.2%) did not continue the treatment. Of those who discontinued the treatment, 11 (36.7%) stopped due to ineffectiveness of the treatment, 7 (23.3%) due to adverse effects, 7 (23.3%) due to bureaucratic challenges in renewing their medical cannabis license, and 5 (16.7%) due to other reasons. Of the patients that stopped using cannabis, five reported withdrawal symptoms such as abdominal pain, restlessness, and low mood.

The assessment of daily activities by IADL and ADL showed different outcomes. The mean ADL scores before and after treatment were 4.4 ± 1.8 and 4.5 ± 1.8, respectively (*p* = 0.27), and the mean IADL scores before and after treatment were 4.1 ± 2.6 and 4.7 ± 3, respectively (*p* = 0.02, Table 2). The mean age of patients that had an improvement in their IADL score was 77.8 ± 6.6 compared to 85.2 ± 7.7 in patients who did not have an improvement in their IADL score (*p* = 0.001). Among those who had an improvement in their IADL score, the median age was 78 (IQR 74–83.8).

Multivariable analysis of the improvement in the different scales showed that age under 80 years was associated with an improvement in the GDS score (odds ratio (OR) 4.5, 95% CI 1.03–19.4) and that male sex was associated with improvements in IADL scores (OR 14.6, 95% CI 1.8–117.7). The type of cannabis strain and the number of times cannabis was used each day were not associated with improvements in the different scores (Table 3).

After six months of cannabis treatment, 63 patients (52.9%) reported stopping the use of at least one chronic medication; of them, 28 (23.5%) stopped using opioid analgesics. Conversely, 24 patients (20.2%) added a new chronic medication, but only 4 (3.4%) added opioids (Table 4). When considering only the patients treated with opioids before cannabis treatment, the mean morphine milligram equivalent (MME) per day before treatment was 49.0 ± 40.7 compared to 27.9 ± 39.1 after treatment (*p* = 0.008).

## 4. Discussion

In this prospective cohort study of older adults treated with medical cannabis, we have shown that cannabis treatment for six months was associated with improvements in IADL and GDS, as well as a reduction in pain and opioid use. No significant changes were found in ADL or in appetite following cannabis treatment.

The effect of cannabis on scales of functional status in older adults was not assessed in previous studies. A study of younger patients, in which only 5% of the population was above 70 years of age, showed that cannabis improved areas of bodily pain, physical functioning, and social functioning, but limitations due to either physical or emotional problems were not changed [24]. Another study of older adults in Colorado and Illinois showed an association between more frequent cannabis use and higher scores on health-related quality of life and healthcare utilization scales [25]. The Lawton IADL include using a telephone, shopping, preparing food, housekeeping, laundry, finance managing, and responsibility for medications [16]. ADL include bathing, dressing, continence, feeding, and toileting [15]. Among older adults, there is a significant difference between age groups, with younger patients having a higher probability of achieving improvement in IADL scores. In spite of this, among those that improved their IADL scores, the median age was 78, i.e., almost half of the patients were over 80 years old. When evaluating the association of age and improvement in ADL or IADL scores in multivariable analysis, there was no significant association to age under 80. On the contrary, there was a significant association in multivariable analysis between male sex and improvement in IADL scores. Emerging research has illuminated intriguing sex-specific patterns in functional decline. While there is a general consensus that women tend to outlive men, studies suggest that this longevity may not necessarily translate to greater functional independence. Some investigations indicate that women may experience a steeper decline in IADL capabilities compared to men, potentially attributed to factors such as hormonal changes and a higher prevalence of certain chronic conditions [26].

Neither the number of times cannabis was used daily nor the THC content of the cannabis were associated with changes in ADL or IADL scores in the multivariable analysis. The minimally important changes for both Katz ADL and Lawton IADL were determined to be around half a point [27]. In our cohort, the mean changes in ADL and IADL scores were 0.1 and 0.6, respectively, before and after cannabis treatment. Therefore, the change in IADL score was clinically significant and probably correlates with the overall satisfaction obtained from the treatment. The mechanism by which cannabis possibly improves the functional status is by improving chronic pain, mood, and general well-being. Another potential mechanism is the reductions in certain drugs, such as benzodiazepines, which have been shown to impact functional status in older adults [28,29]. All these changes enable older adults to function more independently in society, but these changes cannot improve functions such as continence, toileting, or dressing, which are reflected in the ADL scores. Thus, it is not surprising that medical cannabis improves IADL but not ADL.

The main indication for cannabis use in our study was neuropathic pain. This is the most common indication for cannabis in Israel as well as in other countries [11,30,31]. It should be noted that the proportion of patients with malignancies was smaller than expected, but this is due to lack of oncologists in the clinic where the study was conducted. The number of patients with an indication of Parkinson’s disease was also relatively high, as this is indication is found in less than 1% of patients in the general population [31]. This difference is probably attributed to the older age of the population in our study.

The most common route of administration for cannabis in our study was tinctures (cannabis oil). This route of administration is the most preferred for older adults and differs from younger individuals, especially those without a previous experience in smoking tobacco [32]. Consuming cannabis via this route prolongs the effect of cannabis, which is usually suitable for aiding chronic conditions.

The composition of cannabis products was heterogenous among our study group. Most of the patients used either CBD-rich cannabis or cannabis with equal amounts of THC and CBD. This finding is in line with other studies exploring the use of cannabis in older adults [11], and can be attributed to the will of both the prescribing physician and the patients to avoid adverse effects caused by high doses of THC. These adverse effects include gait and balance problems, primarily caused by the effects of THC [12]. Additionally, it is plausible that the anti-inflammatory properties of CBD contribute to the improvement in daily activities [33].

Two distinct patterns can be seen in our results: on the one hand, one-quarter of the patients decided to discontinue the treatment with cannabis after six months; on the other hand, almost all other patients reported that cannabis led to improvement in their overall condition, and two-thirds reported either moderate or significant improvement. These high rates of satisfaction can be explained by the high expectations from cannabis and its perceived efficacy in the general population [11,34]. The relatively large proportion of patients that stopped using cannabis supports the notion that cannabis is not a magical cure that can aid all illnesses.

The effectiveness of cannabis in addressing mood-related disorders and overall quality of life among older individuals has been substantiated through multiple investigations, including our own [10,11,35]. Our present outcomes concur with these findings, illustrating that cannabis contributes to the amelioration of depressive symptoms, as indicated by the GDS score, while also achieving discernible pain alleviation. The average alteration in GDS scores, measured as a change of 1.4 points, surpasses the established threshold of clinical significance, previously determined as 1.2 points [36]. When evaluating the association of age and improvement in GDS scores in multivariable analysis, there was a significant association with age under 80 compared to age above 80 years. However, neither the number of uses of cannabis every day nor the THC content of the cannabis were associated with changes in the GDS score in multivariable analysis.

The reduction in the VAS pain score after cannabis treatment is significant, both statistically and clinically. Clinically significant pain alleviation is characterized by a reduction of 2 points on a 0-to-10 numerical pain rating scale or a noteworthy reduction of 30% in pain intensity [37,38]. We demonstrate a reduction of 3.3 points of the average before and after the treatment. This significant reduction also explains the generally perceived effectiveness of cannabis and the reduction in other medications as the main indication for treatment in our cohort was pain reduction. Despite these remarkable numbers, which were reproduced in previous studies from our group [10], as well as other studies [11], the effectiveness of cannabis in treating chronic pain is still an issue of debate. Recent meta-analyses of randomized controlled trials and observational studies continue to conclude there is only weak evidence for the efficacy of cannabis for the treatment of chronic pain [39,40]. This discrepancy has also been discussed in the literature [41], and is attributed to the difficulty of reproducing cannabis studies between different countries and to the different constituents of cannabis, which are not identical in all studies.

Our findings also demonstrate the relatively favorable safety profile of cannabis therapy, as evidenced by the notably low incidence of adverse events necessitating medical intervention. In addition, less than 10% of the cohort defined the adverse effects as most severe. These observations align with the outcomes of our previous investigations involving older adult cohorts [10,35], as well as analogous comprehensive studies [11]. Dizziness, the most common adverse event in our study, is a well-known adverse event effect of cannabis. The profile of adverse effects was similar in our cohort of older adults compared to cohorts of younger adults treated with cannabis [42].

As evidenced in our prior investigation [10], a noteworthy outcome emerged with the implementation of cannabis treatment, namely a discernible reduction in the utilization of chronic medications. This transformation was particularly pronounced within the realm of analgesics, with opioids, in particular, exhibiting a marked decline in usage. The minimal clinically important difference for a reduction in opioid dosage was determined as a percentage change of 28.2% in the MME [43]. In our cohort, the reduction in opioid use met this criterion, as there was a reduction of 43.0% after cannabis treatment. In addition to this meaningful dose reduction, there was also a significant reduction in the pain VAS scores. Many studies have already assessed the opioid-sparing effect of cannabis, but it should be stated that this effect was also assessed in a recent meta-analysis, which found that the effects remain uncertain due to the very low certainty evidence [25]. We also saw a reduction in benzodiazepines and anti-hypertensives, but in a smaller fashion than with a previous cohort [10].

Cannabis has a known effect on appetite hormones [44,45]; nevertheless, the efficacy of cannabis treatment for improving appetite-related symptoms has not been consistently demonstrated in randomized controlled trials [20]. Our results are consistent with most of these studies, showing no net effect of cannabis on appetite: most patients reported no change and a similar number of patients reported an increase or decrease in appetite.

Our study has several limitations. First, the observational nature of our study can only allow us to determine association and not causality. Second, the study was conducted as a before–after study without a control group. Third, not all participants used the same kind, same dose, and same composition of cannabis. Fourth, most of the patients in the study were treated with cannabis for pain, limiting generalizability for other common indications, such as malignancy.

## 5. Conclusions

Medical cannabis in older adults can improve functional status and mood, as illustrated by better scores in IADL and GDS after six months of treatment. In addition, it is perceived as contributing to the general condition and reduces pain and the use of analgesics, including opioids. It is essential to gather additional evidence-based data, including data from double-blind randomized controlled trials, for this specific population.

## Figures and Tables

**Table 1:** Characteristics of the patients.

Variable	Number of Patients (N = 119)
**Demographic characteristics**	
Age (years, mean ± SD)	79.3 ± 8.5
(median, IQR)	80 (74–86)
Female (n, %)	74 (62.2%)
**Cannabis treatment indications (n, %)**	
Non-specific chronic pain (including neuropathic pain)	57 (47.9%)
Parkinson’s disease	9 (7.6%)
Oncologic treatment	4 (3.4%)
Orthopedic pain	3 (2.5%)
Dementia	2 (1.7%)
Others	6 (5.0%)
**Cannabis treatment data (n, %)**	
Previous cannabis use (n, %)	8 (6.7%)
Route of administration (n, %)	
Tincture	61 (51.3%)
Smoking/Vaporizing	9 (7.6%)
Both tincture and smoking/vaporizing	11 (9.2%)
**Cannabis dosing (n, %)**	
Cannabis administration once a day	10 (8.4%)
Cannabis administration twice a day	17 (14.3%)
Cannabis administration ≥3 day	53 (44.5%)
**CBD and THC composition of cannabis products at treatment initiation (n, %)**	
THC 1%/CBD 20%	5 (4.2%)
THC 3%/CBD 15%	10 (8.4%)
THC 5%/CBD 10%	14 (11.8%)
THC 10%/CBD 2%	11 (9.2%)
THC 10%/CBD 10%	4 (3.4%)
THC 15%/CBD 3%	13 (10.9%)
THC 20%/CBD 1%	2 (1.7%)
**Cannabis adverse events (number of patients, %)**	
Any adverse event	43 (36.1%)
Dizziness	14 (11.8%)
Dry mouth	9 (7.6%)
Psycho-active sensation	9 (7.6%)
Fatigue	7 (5.9%)
Nausea	7 (5.9%)
Drowsiness	5 (4.2%)
Instability	5 (4.2%)
Headache	4 (3.4%)
Heartburn	4 (3.4%)
Constipation	2 (1.7%)
Adverse events defined by patients as most severe	9 (7.6%)
Adverse events requiring medical assistance	2 (1.7%)

**Table 2:** Changes in different scores six months after cannabis treatment.

Score Name	Before Treatment (Mean ± SD)	After Treatment (Mean ± SD)	*p*-Value
Geriatric Depression Scale (GDS) *	6.4 ± 3.9	5 ± 3.9	**0.015**
Katz Activities of Daily Living (ADL) ^†^	4.4 ± 1.8	4.5 ± 1.8	0.268
Lawton Instrumental Activities of Daily Living (IADL) ^‡^	4.1 ± 2.6	4.7 ± 3	**0.023**
Visual Analogue Scale (VAS) for Pain ^§^	8.8 ± 2	5.5 ± 3.2	**<0.001**

* **Geriatric Depression Scale (GDS)**—Scale of 0–15. A score of ≥ 5 suggests depression; higher scores indicate worse depression symptoms. ^†^ **Katz Activities of Daily Living (ADL)**—Scale of 0–6, where 6 = high (patient independent) and 0 = low (patient very dependent). ^‡^ **Lawton Instrumental Activities of Daily Living (IADL)**—Scale of 0–8, where 8 = high function (patient independent) and 0 = low function (patient very dependent). ^§^ **Visual Analogue Scale (VAS) for Pain**—Scale of 0–10, where 0 represents no pain and 10 represents pain as bad as it could possibly be.

**Table 3:** Association of baseline patient characteristics with improvement in different scores after six months of cannabis treatment.

Variable	GDS Odds Ratio (95% CI)	ADL Odds Ratio (95% CI)	IADL Odds Ratio (95% CI)	VAS Odds Ratio (95% CI)
Age under 80 (vs. over 80)	**4.5 (1.03–19.4)**	0.4 (0.1–1.7)	3.2 (0.6–16.1)	0.7 (0.2–2.6)
Male (vs. female)	0.8 (0.2–4.4)	6.7 (0.97–46.3)	**14.6 (1.8–117.7)**	2.5 (0.5–12.2)
Cannabis used twice or less a day (vs. three times a day or more)	1.6 (0.3–9.1)	0.2 (0.03–1.8)	0.3 (0.04–2.6)	1.1 (0.2–6.0)
THC-rich cannabis strain (vs. non-THC-rich cannabis strain)	0.5 (0.1–2.7)	5.8 (0.9–38.5)	1.9 (0.3–13.8)	0.5 (0.1–2.6)

GDS—Geriatric Depression Scale, ADL—Katz Activities of Daily Living, IADL—Lawton Instrumental Activities of Daily Living, VAS—Visual Analogue Scale for Pain.

**Table 4:** Changes in drug regimens after six months of treatment with cannabis.

Drug Class	Number of Patients Who Stopped Using a Certain Drug	Number of Patients Who Reduced the Dose of a Certain Drug	Number of Patients Who Increased the Dose of a Certain Drug	Number of Patients Who Added a New Drug
Opioid analgesics *	28 (23.5%)	1 (0.8%)	1 (0.8%)	4 (3.4%)
Other analgesic drugs ^†^	7 (5.9%)	0 (0%)	0 (0%)	6 (5%)
Benzodiazepines	9 (7.6%)	1 (0.8%)	1 (0.8%)	1 (0.8%)
Neuropathic pain drugs ^‡^	6 (5%)	0 (0%)	0 (0%)	3 (2.5%)
SSRI, SNRI, or NaSSA	7 (5.9%)	3 (2.5%)	0 (0%)	6 (5%)
Antihypertensive drugs	6 (5%)	4 (3.4%)	0 (0%)	4 (3.4%)
Antidiabetic drug	1 (0.8%)	3 (2.5%)	0 (0%)	3 (2.5%)
Anti-psychotics	2 (1.7%)	0 (0%)	0 (0%)	1 (0.8%)
All other drugs	30 (25.2%)	6 (5%)	2 (1.7%)	12 (10.1%)

* Includes: Morphine, tramadol, fentanyl, oxycodone, buprenorphine, oxycodone–naloxone (Targin), acetaminophen-oxycodone (Percocet), codeine–caffeine–paracetamol (Rokacet). ^†^ Includes: NSAIDs (non-steroidal anti-inflammatory Drugs), paracetamol, dipyrone. ^‡^ Includes: Pregabalin, gabapentin, amitriptyline. SSRI—Selective serotonin reuptake inhibitor; SNRI—Serotonin–norepinephrine reuptake inhibitor, NaSSA—Noradrenergic and specific serotonergic antidepressants.

## Data Availability

The data used in the analysis of this study are not publicly available due to the national regulations but are available from the corresponding author upon request.
